# Polygenic risk scores predict blood pressure changes independent of dietary intervention: a secondary analysis of the NUPRESS trial

**DOI:** 10.1007/s00394-026-04072-x

**Published:** 2026-08-01

**Authors:** Luciana C. Holzbach, Aline Marcadenti, Angela C. Bersch-Ferreira, Rachel H. Vieira Machado, Ana Paula P. F. Carvalho, Sônia L. Pinto, Andreza M. Penafort, Alexandre S. G. Coelho, Cristiane Cominetti

**Affiliations:** 1 Nutrition Undergraduate Course, Federal University of Tocantins, Quadra 109 Norte, Av. NS-15, Alcno-14, Bloco Bala I, Plano Diretor Norte, Palmas, TO CEP 77001-090 Brazil; 2 Nutritional Genomics Research Group, School of Nutrition, Federal University of Goiás, Rua 227, s/n, Quadra 68, Leste Universitário, Goiânia, GO CEP 74605080 Brazil; 3 Hcor Research Institute, Syrian Charitable Association (Associação Beneficente Síria), Rua Desembargador Eliseu Guilherme, 200, Paraíso, São Paulo, SP CEP 04003-905 Brazil; 4 Graduation Program in Health Sciences (Cardiology), Cardiology Institute, University Foundation of Cardiology, Av. Princesa Isabel, 395, Santana, Porto Alegre, RS CEP 90040-371 Brazil; 5 Faculty of Medical Sciences, Undergraduate Program in Nutrition, Largo Santa Cecília, 47, Vila Albuquerque, São Paulo, SP CEP 01225-010 Brazil; 6 Portuguese Charity of São Paulo (Beneficência Portuguesa de São Paulo), Rua Maestro Cardim, 769, Bela Vista, São Paulo, SP CEP 01323-900 Brazil; 7 Health and Nutrition Research and Improvement Group, University Hospital, Federal University of Goiás, Rua 235, 285, Quadra 68, Lote Área, Setor Leste Universitário, Goiânia, GO CEP 74605-050 Brazil; 8 Nutrition Undergraduate Course, Fortaleza University, Av. Washington Soares, 1321, Fortaleza, CE CEP 60811-905 Brazil; 9 School of Agronomy, Federal University of Goiás, Av. Esperança, s/n. Campus Samambaia, Goiânia, GO CEP 74690-900 Brazil

**Keywords:** Adults, Genetic risk score, Genetic polymorphisms, Nutrigenetics, Nutritional intervention

## Abstract

**Purpose:**

This study aimed to investigate whether genetic variability, assessed through polygenic risk scores (PRSs), predicts blood pressure (BP) changes in individuals with systemic arterial hypertension (SAH), and to explore potential interactions between genetic risk and dietary interventions.

**Methods:**

This study represents a secondary analysis of data from the Brazilian multicentre trial (NUPRESS), which compared a Dietary Approach to Stop Hypertension (DASH) diet (control group) with a multicomponent intervention (intervention group) over 180 days in adults with SAH. Participants underwent anthropometric assessment, cardiovascular risk evaluation, dietary assessment, and genotyping using a genomic microarray. Single nucleotide polymorphisms (SNPs) associated with BP changes were identified and used to construct PRSs. Multiple linear regression models were employed to examine associations between PRSs, dietary interventions, and changes in systolic (ΔSBP) and diastolic BP (ΔDBP).

**Results:**

A total of 114 SNPs were associated with ΔSBP and 101 with ΔDBP. The resulting PRSs showed strong correlations with both ΔSBP (*r* = 0.85; *p* < 0.0001) and ΔDBP (*r* = 0.84; *p* < 0.0001). No significant effect of dietary intervention on BP changes was observed, and no interaction was identified between PRSs and dietary approach for ΔSBP (*p* = 0.98) or ΔDBP (*p* = 0.13).

**Conclusion:**

PRSs derived from BP-associated SNPs were strong predictors of BP changes over six months, independent of dietary intervention. These findings support the potential utility of genetic profiling in predicting individual variability in BP response, although replication in independent cohorts is warranted. Main study trial registration number NCT03793881 on ClinicalTrials.gov.

**Supplementary information:**

The online version contains supplementary material available at 10.1007/s00394-026-04072-x.

## Introduction

Systemic arterial hypertension (SAH) has a complex aetiology and is strongly influenced by genetic, epigenetic, environmental, and social factors [1, 2].

To reduce the prevalence of SAH, a multi-faceted approach targeting both causal and aggravating factors is necessary. Nutritional interventions, such as the Dietary Approaches to Stop Hypertension (DASH), have emerged as effective strategies [3, 4]. This diet emphasizes reduced sodium intake, increased potassium intake, and the consumption of low-fat dairy products to control blood pressure (BP) [1, 5, 6].

Integrative approaches, like mindfulness-based stress reduction, have proven effective in lowering BP for individuals with SAH or prehypertension [7, 8]. Self-care strategies, such as medication adherence, stress and depression management, weight loss, healthy eating, and increased physical activity, can also contribute to BP control [9–11].

Genome-wide association studies (GWAS) have identified between 65 and 901 genetic loci associated with BP levels, many lacking established biological connections. Family studies estimate heritability for BP at 24–50% [12, 13], while GWAS-identified loci collectively account for approximately 2% of this heritability [14].

Technological advancements have enabled the identification of over 30 genes and 1,400 single nucleotide polymorphisms (SNPs) associated with BP regulation [15]. However, the individual effect of each SNP is often too small to be statistically significant. This is due to the complex interplay of numerous genetic variants, each contributing a small effect to the overall phenotype. To better understand and predict individual risk, polygenic risk scores (PRSs) have emerged as valuable tools. By aggregating the effects of multiple SNPs identified through GWAS, PRSs enable a more comprehensive assessment of genetic predisposition to SAH and related cardiometabolic conditions [16, 17]. While extensive research has explored the links between genotypes and SAH, studies investigating the interaction between specific dietary interventions and genetic variations on BP responses are limited. Moreover, a PRS that accurately predicts individual BP changes in response to different nutritional approaches remains elusive.

Therefore, hypothesising that genetic factors influence the effectiveness of nutritional interventions for SAH, this study aimed to investigate whether genetic variability, assessed through PRSs, predicts BP changes in individuals with SAH, and to explore potential interactions between genetic risk and dietary interventions.

## Methods

### Study design, participants, and ethical issues

This study was a secondary analysis of data from the multicentre randomized clinical trial “Effectiveness of a two-component nutritional strategy for blood pressure control in individuals with hypertension users of a public health system: a randomized controlled clinical trial” (NUPRESS; ClinicalTrials.gov: NCT03793881). The NUPRESS trial had the primary objective of comparing two nutritional interventions for BP control and followed patients with uncontrolled SAH for six months. Participants were randomized, stratified by research centre, to receive either a multicomponent intervention (intervention group) or a DASH diet (control group) [18]. For the purposes of the present analysis, BP changes over time (systolic BP [ΔSBP] and diastolic BP [ΔDBP]) were used as outcome variables and were analysed in relation to both intervention group and genetic profile.

The NUPRESS study included patients from geographically diverse centres across five Brazilian regions, coordinated by the Hcor Research Institute. Inclusion criteria were age ≥ 21 years, diagnosis of SAH, no prior nutritional guidance in the past six months, and SBP ≥ 140 mmHg at screening. Exclusion criteria were as previously described [18].

This secondary study was conducted in a subset of participants for whom blood samples were available in a biorepository, allowing genotyping and PRSs construction.

Participants in the NUPRESS trial were randomized in a 1:1 ratio to either a multicomponent intervention group or a DASH diet control group, using centralized randomization procedures after eligibility confirmation. The interventions have been previously described in detail [18]. Briefly, the control group received dietary counselling based on the DASH diet. The intervention group received a multicomponent program combining two main domains: (1) nutritional counselling based on the Dietary Guidelines for the Brazilian Population [19], and (2) behavioural strategies aimed at promoting sustainable lifestyle changes. These behavioural components included mindfulness-based approaches, stress management techniques, and support for self-care and goal setting.

Both groups were followed for six months, with periodic in-person visits and remote support to reinforce adherence. BP and other clinical and anthropometric variables were assessed longitudinally, as previously described [18].

This secondary study was approved by the Research Ethics Committee of the Federal University of Goiás (Protocol No. 4,584,806), the Research Ethics Committee of the Federal University of Tocantins (Protocol No. 5,435,511), the Hcor Research Institute (Protocol No. 5,642,195), and the National Research Ethics Committee (Protocol No. 5,922,586).

### Anthropometry, lifestyle, and food consumption

This secondary study utilised anthropometric, lifestyle, and food consumption data collected during the NUPRESS study. Anthropometric measurements included weight, height, and waist circumference (WC), obtained following standardized protocols [20].

Sociodemographic data, including social class and level of education, were collected using a questionnaire developed by the Brazilian Association of Research Companies [21]. Physical activity levels were assessed using the short version of the International Physical Activity Questionnaire (IPAQ), adapted and validated for the Brazilian population [22].

Dietary intake was assessed using 24-hour dietary recalls (24-h recalls) collected at baseline (visit 1) and at the end of the intervention period (visit 5). Two 24-h recalls were collected at each time point: one during the in-person visit and one via telephone within 7 days. This approach is commonly recommended to better capture day-to-day variability in dietary intake while reducing participant burden. The Multiple Pass Method (MPM) was employed for all 24-h recalls [23]. Dietary data were entered and analysed using the Vivanda^®^ software, a nutritional analysis software based on Brazilian food composition tables and routinely used in Brazil for dietary assessment and nutrient composition analysis. At both baseline and the end of the intervention, dietary intake was estimated as the mean of two 24-h recalls: one collected during the study visit and a second collected by telephone within the following 7 days. Similarly, the final dietary intake was assessed by averaging the R24 h obtained at the final visit with a corresponding telephone-administered 24 h also applied within 7 days of the final visit. To adjust for variations in energy intake, consumption of nutrients was energy-adjusted using linear regression analysis. The following dietary variables were evaluated: energy (kcal/day), protein (g/day and % of total energy – TE), lipid (g/day and %TE), saturated fatty acid (SFA) (g/day and %TE), cholesterol (mg/day), sodium (mg/day), fibre (g/day), calcium (mg/day), potassium intake (mg/day), and magnesium (mg/day).

### Blood pressure

BP was assessed using an automated arm BP monitor (G-Tech model BP3BK1–3, Shenzhen, China) during each patient visit to the participating centres. Mean SBP and DBP values were recorded according to the guidelines of the Brazilian Society of Cardiology (SBC) [1].

### Genetic markers

Genomic DNA was extracted from whole blood samples using the PureLink^®^ Genomic DNA kit (Invitrogen, Waltham, USA) following the manufacturer’s protocol. This involved proteinase K and RNase A digestion at 56 °C for 35 min with shaking, followed by ethanol precipitation and DNA binding to a silica column. After washing, the DNA was eluted in 200 µL of ultrapure water at 56 °C for 10 min. DNA purity was assessed by spectrophotometric measurement of the 260/280 nm absorbance ratio using a NanoDrop^TM^1000 spectrophotometer (Thermo Fisher, Waltham, USA), and DNA concentration was determined based on the absorbance at 260 nm.

Genotyping was performed using the Axiom™ Precision Medicine Diversity Research Array (catalogue number 951962; Applied Biosystems, Waltham, USA) on a GeneTitan platform (catalogue number 00-0372). This array contains approximately 800,000 SNP probes. Genotyping procedures followed the manufacturer’s instructions. Data were analysed using Axiom Analyses Suite Software 5.2 (Thermo Fisher, Waltham, USA).

SNPs were selected based on quality control criteria: a call rate ≥ 97% and a Dish Quality Control (DQC) score ≥ 0.82. SNPs were classified into categories such as Poly High Resolution, No Minor Hom, Mono High Resolution, Other MA, Off-Target Variants (OTV), and Call Rate Below Threshold. Only SNPs classified as “Poly High Resolution” and “No Minor Hom” were included in the subsequent analyses.

Candidate SNPs were identified through a structured literature-based approach. This included: (1) a scoping review of SNPs associated with BP responses to nutritional interventions [24]; (2) studies investigating genetic variants associated with SAH in the Brazilian population; and (3) GWAS related to BP regulation. The SNPs identified from these sources were then cross-referenced with those available on the genomic microarray. When a candidate SNP was not directly represented on the array, a proxy SNP located within a 200 kb window was selected based on genomic proximity and linkage disequilibrium (LD) patterns, following established approaches in genetic association studies and genotype imputation methods [25, 26].

This process resulted in a set of SNPs available for analysis, which were subsequently tested for association with ΔSBP and ΔDBP.

### Statistical analysis

Data from the NUPRESS study used in this secondary study were double-entered into Excel^®^ sheets for data quality control. Anthropometric, dietary, and clinical data were summarized using descriptive statistics. Mean values and 95% confidence intervals (95% CI) were calculated for continuous variables. The unpaired Student’s t-test was used to compare between-group differences (intervention vs. control), while paired t-tests were used to assess within-group changes over time. Sociodemographic variables, including socioeconomic classification, education, and marital status were summarized using frequencies and percentages. Group comparisons for categorical variables were performed using the chi-square test or Fisher’s exact test, as appropriate.

To ensure data quality, the adherence of each SNP to Hardy-Weinberg Equilibrium (HWE) was checked using Pearson’s chi-square test based on individual allele frequencies. SNPs that significantly deviated from HWE were excluded from further analyses.

To assess the differential effects of the DASH diet and the multicomponent intervention on BP, linear regression models were employed. The dependent variables included SBP, DBP, ΔSBP, and ΔDBP. Potential confounders, including age, sex, race, education level, socioeconomic status, BMI, WC, and antihypertensive medication use, were included as covariates in the initial regression models. A backward stepwise selection procedure was utilized to identify the most parsimonious model, with the final model selection based on the Akaike Information Criterion (AIC).

The ΔSBP and ΔDBP were calculated by subtracting the baseline values from the final values. These changes were then adjusted for the effects of significant independent variables. When no significant differences in BP were observed between the intervention and control groups, the data from both groups were combined for subsequent analyses.

For SNPs with missing allele frequency data, imputation was performed using the mean value within the genotype table. To identify SNPs associated with BP outcomes, each SNP was individually tested using linear regression analysis. This included SNPs identified in the literature review and those present on the genotyping array, as well as proxy SNPs within a 200 kb window for those not directly represented on the array. A significance level of 0.10 was used for these analyses.

Significant SNPs were then used to build PRSs, which were constructed as weighted scores, calculated as the sum of risk alleles for each SNP multiplied by their respective regression coefficients (β) obtained from the individual SNP association analyses. Thus, the β coefficients represent the estimated effect size of each SNP on BP outcomes and were used to weigh their contribution to the overall PRS. Because β coefficients were derived from regression models including the same outcome variables, they allow comparison of the relative contribution of each SNP within the PRS. The initial PRSs were further refined using a backward stepwise selection procedure to retain the most informative genetic variants contributing to BP outcomes. Finally, the associations between the refined PRSs and BP outcomes were assessed using Pearson’s correlation analysis. Linear regression models were subsequently employed to investigate the potential interaction between the type of nutritional intervention (DASH diet vs. multicomponent intervention) and the PRSs on BP outcomes.

All statistical analyses were performed using R software (version 4.3.1), including power analysis calculations using the ‘pwr’ package [27]. Power analysis was conducted for a multiple linear regression model, assuming a mean effect size of 0.15 [28], a Type I error rate of 0.05, and a sample size of 224 participants. The estimated statistical power was 0.993.

## Results

**Fig. 1:**
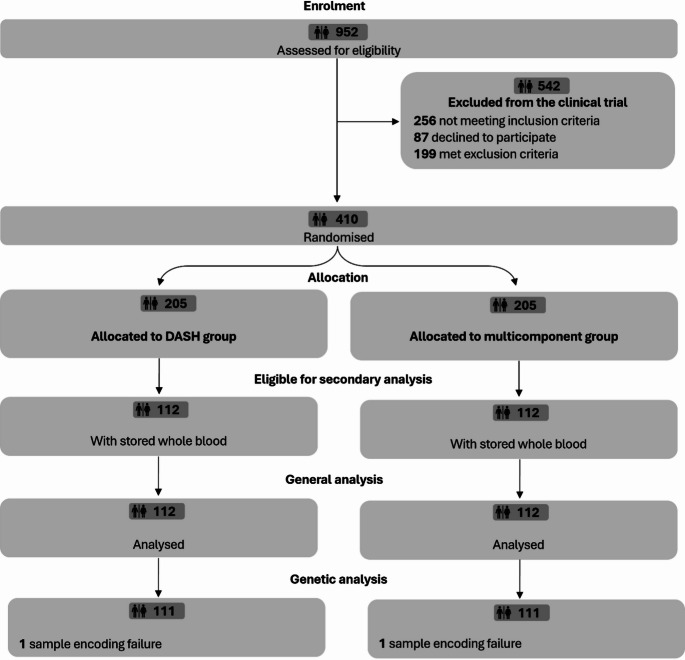
Participant selection flowchart According with CONSORT flowchart [29]

A total of 224 participants from the NUPRESS trial met the eligibility criteria for this secondary analysis and were equally allocated to the DASH (*n* = 112) and multicomponent intervention (*n* = 112) groups (Fig. 1). The mean age was 50.4 ± 11.0 years, and the majority of participants were female (61.2%), married (58.5%), and had more than a high school education (70.5%). Additional demographic and socioeconomic characteristics are presented in Table 1.

Over the 6-month follow-up period, modest changes in BP were observed, with no significant differences between the intervention groups (Table 2). Given the absence of differential effects of dietary interventions, subsequent analyses were conducted to investigate whether genetic variability could explain interindividual differences in BP response.

Accordingly, genetic analyses were performed to identify SNPs associated with changes in SBP and DBP, and to construct PRSs to evaluate their predictive value.

**Table 1:** Demographic and socioeconomic characteristics of participants

Variables		Total (*n* = 224)	DASH (*n* = 112)	Multicomponent (*n* = 112)	*p*-value
Age (years)		50.4 ± 11.0	50.7 ± 11.5	50.2 ± 10.6	0.76
Sex	Female	137 (61.2%)	48 (42.9%)	39 (34.8%)	0.22
	Male	87 (38.8%)	64 (57.1%)	73 (65.2%)	
Marital status	Married	131 (58.5%)	67 (59.8%)	64 (57.1%)	0.97
	Divorced	23 (10.3%)	10 (8.9%)	13 (11.6%)	
	Single	39 (17.4%)	19 (17.0%)	20 (17.9%)	
	Common-law marriage	20 (8.9%)	10 (8.9%)	10 (8.9%)	
	Widowed	11 (4.9%)	6 (5.4%)	5 (4.5%)	
Education level	I	26 (11.6%)	16 (14.3%)	10 (8.9%)	0.62
	II	20 (8.9%)	8 (7.1%)	12 (10.7%)	
	III	20 (8.9%)	9 (8.0%)	11 (9.8%)	
	IV	85 (37.9%)	41 (36.6%)	44 (39.3%)	
	V	73 (32.6%)	38 (33.9%)	35 (31.3%)	
Mean monthly income (US$)^a^	1.973–1.331	9 (4.0%)	4 (3.6%)	5 (4.5%)	0.69
	705	13 (5.8%)	8 (7.1%)	5 (4.5%)	
	408	47 (21.0%)	25 (22.3%)	22 (19.6%)	
	242	58 (25.9%)	32 (28.6%)	26 (23.2%)	
	147	59 (26.3%)	25 (22.3%)	34 (30.4%)	
	98–56	38 (17.0%)	18 (16.1%)	20 (17.9%)	
Physical activity level	High	38 (17.0%)	15 (15.2%)	21 (18.8%)	0.68
	Moderate	48 (21.4%)	26 (23.2%)	22 (19.6%)	
	Low	138 (61.6%)	69 (61.6%)	69 (61.6%)	

Data are presented as absolute and relative frequencies [n (%)]. Education level: I—Illiterate/Incomplete Elementary School; II—Complete Elementary School I/Incomplete II; III—Complete Elementary School II/Incomplete High School; IV—Complete High School/Incomplete Higher Education; V—Complete Higher Education.* P*-value obtained by Student’s t-test, Fisher’s exact test or Pearson’s χ^2^ test. ^a^ 1 US$ = R$4.93 Brazilian reais.

### Blood pressure and anthropometric outcomes

At baseline, SBP and DBP did not differ significantly between the DASH and multicomponent intervention groups. Over the 6-month follow-up, modest reductions in SBP were observed in the overall sample, decreasing from 139.6 (137.0–142.1) mmHg to 134.7 (132.3–137.1) mmHg (*p* = 0.006). In contrast, DBP did not change significantly over time [90.9 (89.4–92.5) mmHg vs. 89.2 (87.3–91.2) mmHg; *p* = 0.18] (Table 2).

In multivariable linear regression models, the type of nutritional intervention was not associated with ΔSBP and ΔDBP. For ΔSBP, only age remained a significant predictor (β = −0.42, *p* < 0.001; r² = 0.05). For ΔDBP, age (β = −0.25, *p* = 0.003), sex (β = 3.68, *p* = 0.08), physical activity level (β = 0.0006, *p* = 0.07), and WC (β = −0.17, *p* = 0.05) were retained in the final model (r² = 0.06). After adjustment for these variables, no significant differences were observed between the DASH and multicomponent groups in ΔSBP (*p* = 0.41) or ΔDBP (*p* = 0.34).

Given the absence of an effect of the interventions on BP changes, participants were analysed as a single group in subsequent genetic analyses.

No significant differences were observed between the DASH and multicomponent groups for weight, BMI, or WC at baseline or at the end of the intervention. Similarly, no significant changes were observed in anthropometric measures in the total sample over the 6-month follow-up (Table 2).

### Dietary intake changes

Dietary intake analyses were included to verify that participants modified their dietary patterns during follow-up, thereby confirming adherence to the nutritional interventions. Overall, participants reported modest but consistent improvements in dietary intake during follow-up. Daily TE decreased by approximately 13%, accompanied by reductions in sodium intake, saturated fat intake, and total fat intake. Although absolute protein intake (g/day) decreased in parallel with the reduction in TE intake, the proportion of energy derived from protein increased, indicating an improvement in overall dietary composition (Table 3).

**Table 2:** Comparison of anthropometric and blood pressure data of participants at the baseline and end of follow-up

Time	Baseline			End			Baseline	End	
Variable	DASH (*n* = 112)	MC (*n* = 112)	*p*-value	DASH (*n* = 103)	MC (*n* = 91)	*p*-value	*n* = 224	*n* = 194	*p*-value
Weight (kg)	83.2 (80.0–86.4)	82.4 (79.4–85.5)	0.72	82.9 (79.7–86.1)	82.0 (78.5–85.5)	0.69	82.8 (80.6–85.0)	82.5 (80.2–84.8)	0.84
BMI (kg/m^2^)	30.9 (30.0–31.8)	31.2 (30.4–32.1)	0.60	30.6 (29.6–31.5)	31.0 (30.0–31.9)	0.59	31.1 (30.5–31.7)	30.8 (30.1–31.4)	0.49
WC (cm)	100.6 (98.5–102.8)	100.2 (97.9–102.4)	0.78	98.8 (95.4–102.1)	101.4 (97.6–105.1)	0.29	100.4 (98.9–101.9)	100.1 (97.6–102.6)	0.85
SBP (mmHg)	139.8 (135.9–143.7)	139.3 (135.8–142.8)	0.84	133.7 (130.2–137.2)	135.8 (132.6–139.0)	0.37	139.6 (137.0–142.1)	134.7 (132.3–137.1)	0.006
DBP (mmHg)	90.7 (88.6–92.9)	91.12 (88.9–93.4)	0.80	88.3 (85.5–91.1)	90.29 (87.5–93.5)	0.30	90.9 (89.4–92.5)	89.2 (87.3–91.2)	0.18

Data expressed as mean and 95% confidence interval. MC (multicomponent); BMI (body mass index); WC (waist circumference); SBP (systolic blood pressure); DBP (diastolic blood pressure).* P*-values obtained by Student’s t-test.

**Table 3:** Comparison of participants’ food consumption data at the baseline and end of follow-up

Variable	Baseline (*n* = 219)	End (*n* = 205)	*p*-value
Energy (kcal/day)	1583.0 (1504.2–1661.8)	1375.8 (1311.2–1440.4)	< 0.0001
Protein (g/day)	73.1 (70.7–75.4)	69.4 (67.2–71.6)	0.02
Protein (%TE)	18.4 (17.8–18.9)	20.2 (19.6–20.8)	< 0.0001
Lipids (g/day)	58.1 (56.3–60.0)	47.0 (45.5–48.6)	< 0.0001
Lipids (%TE)	32.5 (31.5–33.5)	30.8 (29.8–31.8)	0.019
SFA (g/day)	20.0 (19.3–20.7)	15.9 (15.2–16.6)	< 0.0001
SFA (%TE)	11.1 (10.7–11.5)	10.3 (9.8–10.7)	0.01
Cholesterol (mg/day)	269.9 (254.2–285.5)	252.9 (237.2–268.6)	0.13
Sodium (mg/day)	2793.1 (2693.5–2892.7)	2332.3 (2251.7–2412.8)	< 0.0001
Fiber (g/day)	18.6 (17.6–19.7)	17.6 (16.7–18.5)	0.15
Calcium (mg/day)	410.4 (382.5–438.2)	410.6 (381.7–439.5)	0.99
Potassium (mg/day)	1861.6 (1795.9–1927.3)	1870.4 (1806.5–1934.2)	0.85
Magnesium (mg/day)	172.9 (166.0–179.8)	171.8 (165.5–178.1)	0.81

Data expressed as mean and 95% confidence interval. BMI (body mass index); WC (waist circumference); SFA (saturated fatty acids); TE (total energy).* P*-values obtained by Student’s T-test.

### Genotyping and quality control

Genotyping of 868,298 markers resulted in 855,657 high-quality SNPs (call rate ≥ 98.5%; sample call rate 99.6%). After filtering for minor allele frequencies (MAF) (< 0.025), mitochondrial, sex chromosome, and contig SNPs, 441,629 SNPs remained for analysis.

Genetic substructure was assessed and showed no significant ancestry effects; principal components were tested during model refinement but were not retained in the final models.

Two samples were excluded due to insufficient genotyping quality, resulting in a final sample of 222 participants.

### SNP selection and PRSs construction

Candidate SNPs were identified from the literature, including studies on SAH and BP regulation, prioritizing findings from Brazilian and multi-ethnic populations. SNPs identified exclusively in East Asian populations were excluded due to limited representation in the study sample (**Table ****S1**).

A total of 1262 candidate SNPs were identified. Of these, 249 (19.7%) were directly available on the genotyping array. For SNPs not present on the array, proxy SNPs were identified based on genomic proximity and LD, resulting in a total of 1155 SNPs available for analysis.

Each SNP was individually tested for association with ΔSBP and ΔDBP using linear regression. SNPs meeting the predefined significance threshold (*p* ≤ 0.10) were included in the construction of PRSs.

PRSs were calculated as weighted scores, defined as the sum of risk alleles for each SNP multiplied by their respective regression coefficients (β), representing the estimated effect size of each SNP on BP outcomes.

### Association between PRSs and BP outcomes

**Fig. 2:**
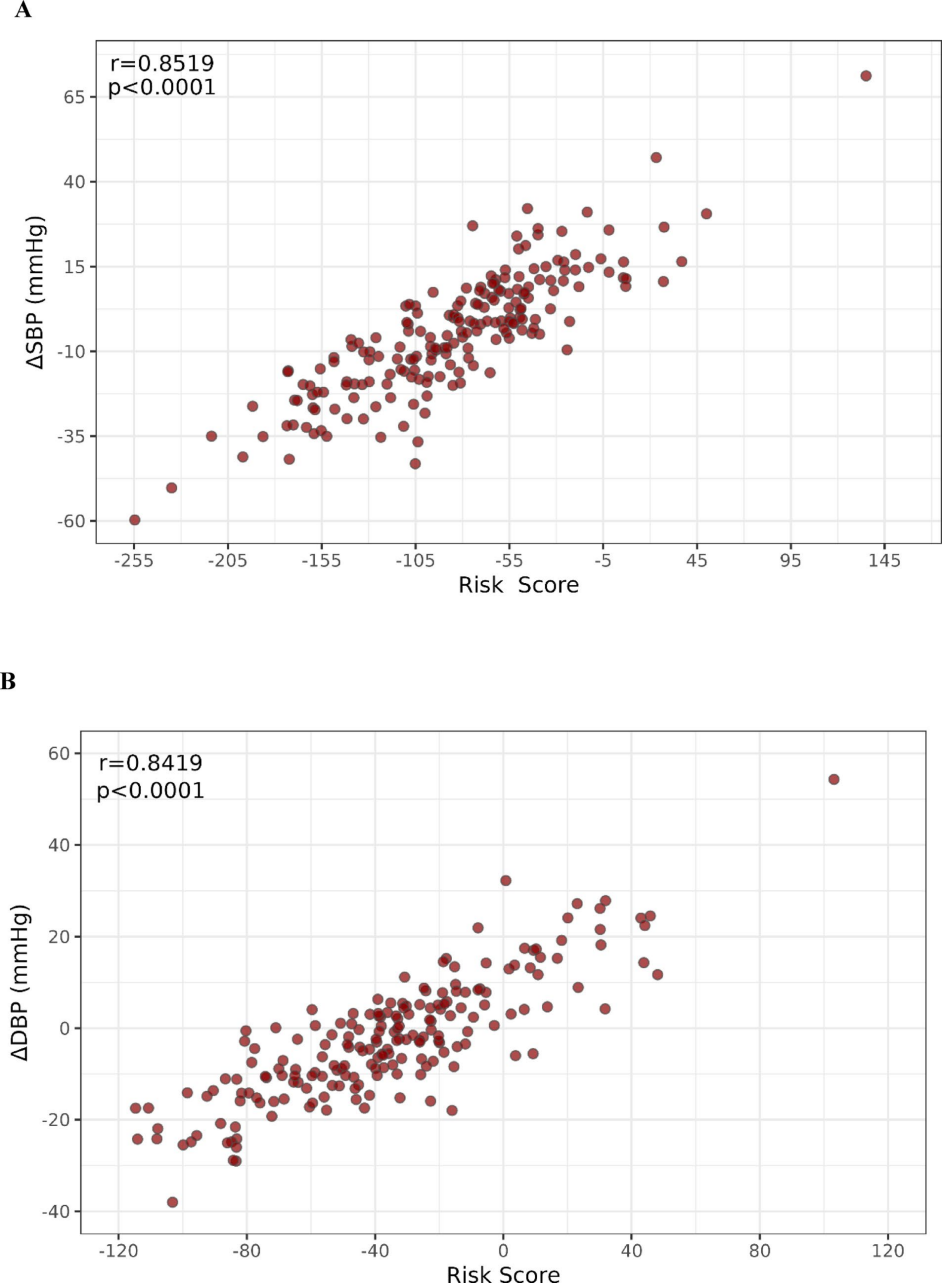
Correlation between PRSs and variations in SBP and DBP after nutritional intervention in patients with systemic arterial hypertension: **A** Association between the constructed PRSs and the variation in systolic blood pressure (∆SBP). **B** Association between the constructed PRSs and the variation in diastolic blood pressure (∆DBP).

The PRS for ΔSBP included 114 SNPs, and the PRS for ΔDBP included 101 SNPs (**Table ****S2**). Both PRSs showed strong correlations with their respective outcomes (ΔSBP: *r* = 0.85, *p* < 0.0001; ΔDBP: *r* = 0.84, *p* < 0.0001), indicating that higher PRSs were associated with greater increases in BP (Fig. 2).

To assess whether dietary intervention modified these associations, PRSs were included in linear regression models with interaction terms. No significant interaction between PRSs and type of nutritional intervention was observed for ΔSBP (*p* = 0.98) or ΔDBP (*p* = 0.13).

## Discussion

In this secondary analysis of the NUPRESS trial, we found that PRSs were strongly associated with interindividual variability in BP changes over a 6-month period. Individuals with higher PRSs values exhibited greater increases in both SBP and DBP, indicating a substantial contribution of genetic factors to BP response. In contrast, no significant differences in BP changes were observed between dietary intervention groups, and no interaction was identified between PRSs and intervention type. Together, these findings suggest that genetic variability may play a more prominent role than the type of dietary intervention in determining BP response in this population.

GWAS have highlighted the complex and polygenic nature of SAH, in which numerous genetic variants with small individual effects interact with environmental factors [30]. In a large study including more than one million individuals, Evangelou et al. identified 901 loci associated with SBP, and developed a PRS that explained approximately 5.7% of the variance in systolic BP [16].

In the Brazilian population, PRSs incorporating varying numbers of SNPs—from a few variants to hundreds of thousands—have also been explored, with heterogeneous predictive performance. For example, a PRS based on 423,000 variants was associated with higher SBP in individuals at the highest genetic risk, whereas models using smaller sets of selected variants showed more limited predictive ability [31]. Conversely, simpler models based on a small number of SNPs have also demonstrated associations with BP traits [32], highlighting the lack of consensus regarding the optimal approach for PRS construction.

It is important to note that most previously published PRSs were developed to predict HAS susceptibility or baseline BP. In contrast, the PRSs developed in the present study were specifically designed to predict interindividual variation in BP response following nutritional interventions, representing a distinct phenotype and a different potential application. In this context, our findings showed stronger associations between genetic variants and BP changes than typically reported for baseline BP measurements. This may be explained by the use of BP variation following a controlled intervention as the phenotype, which may better capture interindividual responsiveness than single time-point measurements. Given that BP is influenced by many variants with modest effects [15], this approach may enhance the detection of genetic contributions to BP dynamics.

Neither the nutritional interventions nor their interaction with genetic profiles significantly influenced BP changes in this study. However, participants showed improvements in dietary intake during follow-up, including reduced energy, saturated fat, and sodium intake, as well as an increased proportion of protein intake, consistent with current dietary recommendations for SAH [1, 3, 4, 6].

Although higher protein intake has been associated with lower BP in previous studies [33, 34], differences in assessment methods and the relatively moderate protein intake observed in our sample (~ 20% of TE) may partly explain the lack of association with BP changes. Similarly, despite the well-established role of sodium reduction in BP control, the magnitude and duration of dietary changes in this study may not have been sufficient to produce measurable differences in BP outcomes. These findings reinforce that, within the context of modest dietary changes, genetic factors may play a more prominent role in explaining interindividual variability in BP response.

Although BP regulation is influenced by multiple genetic variants with small individual effects, a subset of SNPs in our analysis showed stronger associations with BP changes. Among these, rs2384550 demonstrated the most consistent association with both ΔSBP and ΔDBP, with effect sizes suggesting a potentially meaningful contribution to BP variability. This SNP is located near the *TBX3* and *TBX5* genes and has been previously associated with BP traits in African and African American populations [35–37], supporting the robustness of this finding across different cohorts.

Among SNPs previously investigated in Brazilian populations, only the association of *ADRA1A* rs1048101 was replicated in our study. The G allele was associated with greater increases in ΔSBP, consistent with prior evidence linking this variant to SAH risk in individuals over 45 years of age [38]. Although this finding requires replication in independent populations, it suggests that genetic variation in sympathetic vascular regulation may contribute not only to HAS susceptibility but also to interindividual variability in BP response to lifestyle interventions. This result supports the inclusion of *ADRA1A* among candidate variants for future studies investigating the genetic determinants of BP response to nutritional interventions.

We also identified an association between rs17477177 (near *CCDC71L*) and ΔSBP, potentially implicating the neighbouring *PIK3CG* gene, which has been linked to cardiovascular regulation in experimental models [39]. Although the functional relevance of this locus to BP regulation remains to be established, this finding suggests that variants in this genomic region warrant further investigation in studies of BP response to nutritional interventions.

Finally, the *IL6* rs1800795 polymorphism showed associations with BP changes that differed from those reported in other populations, such as Finnish cohorts [40], reinforcing the context-dependent nature of genetic effects on BP regulation.

Overall, these findings illustrate the heterogeneity of genetic influences on BP and underscore the need for replication in diverse populations.

The growing interest in personalized nutrition highlights the importance of understanding interindividual variability in response to dietary interventions. In this context, our findings suggest that PRSs may help explain differences in BP responses beyond the effects of dietary strategies alone.

Although the PRSs developed in this study showed strong associations with BP changes, their application in clinical practice requires caution. Validation in independent cohorts and across different populations is necessary before their use in guiding personalized dietary recommendations.

This study has several limitations, including the limited representation of SAH-associated SNPs on the genotyping array, the use of a convenience sample, and the potential for measurement error in dietary intake assessment. In addition, the absence of external validation restricts the generalizability of the findings. Despite these limitations, this study has important strengths, including the use of a well-characterized clinical cohort with longitudinal BP measurements, the integration of genomic and phenotypic data, and the inclusion of participants from multiple regions of Brazil, which increases the relevance of the findings for diverse populations.

In conclusion, while the type of nutritional intervention did not influence BP response or interact with genetic profile, PRSs derived from BP-associated SNPs were strong predictors of BP variation over time. These results support the role of genetic factors in modulating BP response and highlight the need for further research to validate and refine these approaches in diverse populations.

## Supplementary information

Below is the link to the electronic supplementary material.

Supplementary Material 1

Supplementary Material 2

Supplementary Material 3

## Data Availability

The datasets generated and/or analysed during the secondary study are not publicly available due to privacy and ethical restrictions but are available from the corresponding author on reasonable request, subject to approval by the relevant ethics committee and applicable data protection regulations.
