# Supplementary material for: Polygenic risk scores predict blood pressure changes independent of dietary intervention: a secondary analysis of the NUPRESS trial
Source: Eur J Nutr. 2026 Aug 1;65(5):225. doi: 10.1007/s00394-026-04072-x (PMC13428793; doi:10.1007/s00394-026-04072-x)
Supplement: Supplementary file 1 — Supplementary Material 1 [file 394_2026_4072_MOESM1_ESM.docx]

**Article title:** Polygenic risk scores predict blood pressure changes independent of dietary intervention: a secondary analysis of the NUPRESS trial

**Journal name:** European Journal of Nutrition

**Authors:** Luciana C Holzbach^a,b^, Aline Marcadenti^c,d,e^, Angela C Bersch-Fereira^f^, Rachel H Vieira Machado^c^, Ana Paula P F Carvalho^g^, Sônia L Pinto^1^, Andreza M Penafort^h^, Alexandre S G Coelho^i^, Cristiane Cominetti^b*^

***Corresponding author:** Cristiane Cominetti. Nutritional Genomics Research Group, School of Nutrition. Federal University of Goiás. Rua 227, s/n, Quadra 68, Leste Universitário, CEP 74605080, Goiânia, GO, Brazil. Phone: +55-62-32096270 ext. 210. Fax: + 55-62-32096273. e-mail: [ccominetti@ufg.br](mailto:ccominetti@ufg.br)


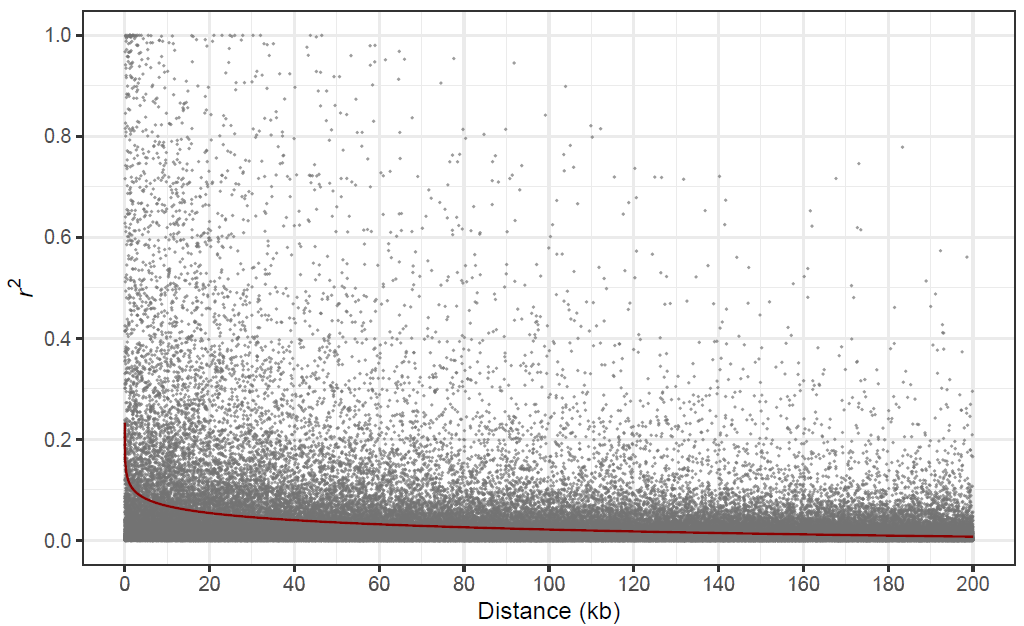


**Fig S1** Linkage disequilibrium decay curve. Linkage disequilibrium decay using inter-SNP comparisons up to 200 Kb.
