# Supplementary material for: Polygenic risk scores predict blood pressure changes independent of dietary intervention: a secondary analysis of the NUPRESS trial
Source: Eur J Nutr. 2026 Aug 1;65(5):225. doi: 10.1007/s00394-026-04072-x (PMC13428793; doi:10.1007/s00394-026-04072-x)
Supplement: Supplementary file 2 — Supplementary Material 2 [file 394_2026_4072_MOESM2_ESM.docx]

**Article title:** Polygenic risk scores predict blood pressure changes independent of dietary intervention: a secondary analysis of the NUPRESS trial

**Journal name:** European Journal of Nutrition

**Authors:** Luciana C Holzbach^a,b^, Aline Marcadenti^c,d,e^, Angela C Bersch-Fereira^f^, Rachel H Vieira Machado^c^, Ana Paula P F Carvalho^g^, Sônia L Pinto^1^, Andreza M Penafort^h^, Alexandre S G Coelho^i^, Cristiane Cominetti^b*^

***Corresponding author:** Cristiane Cominetti. Nutritional Genomics Research Group, School of Nutrition. Federal University of Goiás. Rua 227, s/n, Quadra 68, Leste Universitário, CEP 74605080, Goiânia, GO, Brazil. Phone: +55-62-32096270 ext. 210. Fax: + 55-62-32096273. e-mail: [ccominetti@ufg.br](mailto:ccominetti@ufg.br)

**Table S1** List of SNPs associated with systemic arterial hypertension reported in the literature and used to construct polygenic risk scores.

| rs669 | rs217727 | rs633185 | rs891511 | rs1171096 | rs1607644 |
| --- | --- | --- | --- | --- | --- |
| rs699 | rs223361 | rs646153 | rs894344 | rs1173771 | rs1630736 |
| rs3175 | rs231708 | rs647152 | rs900145 | rs1215469 | rs1650911 |
| rs4308 | rs246973 | rs661348 | rs912434 | rs1220128 | rs1694068 |
| rs4673 | rs258494 | rs668459 | rs917275 | rs1232482 | rs1706003 |
| rs4880 | rs260508 | rs680515 | rs918466 | rs1250129 | rs1718845 |
| rs4961 | rs262986 | rs687621 | rs919045 | rs1250247 | rs1722886 |
| rs5068 | rs267517 | rs694739 | rs925484 | rs1250259 | rs1764975 |
| rs5069 | rs286809 | rs702395 | rs926552 | rs1261744 | rs1799752 |
| rs5186 | rs296797 | rs704191 | rs932764 | rs1263671 | rs1783521 |
| rs5219 | rs319690 | rs709209 | rs953492 | rs1265842 | rs1799837 |
| rs5351 | rs337100 | rs709668 | rs954767 | rs1271309 | rs1799945 |
| rs5370 | rs342989 | rs729448 | rs956006 | rs1275988 | rs1799983 |
| rs5417 | rs347591 | rs729639 | rs963920 | rs1277463 | rs1800470 |
| rs5441 | rs381815 | rs731681 | rs1024323 | rs1321162 | rs1800562 |
| rs5443 | rs385437 | rs737721 | rs1004467 | rs1322639 | rs1800795 |
| rs6271 | rs391578 | rs740406 | rs1004558 | rs1327235 | rs1800796 |
| rs7211 | rs394112 | rs740698 | rs1008058 | rs1331012 | rs1800896 |
| rs7212 | rs405884 | rs743757 | rs1011018 | rs1332813 | rs1801058 |
| rs7255 | rs406222 | rs745821 | rs1012089 | rs1334576 | rs1801133 |
| rs7412 | rs409558 | rs751984 | rs1036477 | rs1344653 | rs1801253 |
| rs7562 | rs415695 | rs757081 | rs1036902 | rs1345198 | rs1801278 |
| rs8258 | rs419076 | rs783621 | rs1042010 | rs1347345 | rs1813353 |
| rs8904 | rs449789 | rs804605 | rs1043069 | rs1350100 | rs1821002 |
| rs12979 | rs452036 | rs804606 | rs1044822 | rs1375564 | rs1821295 |
| rs13303 | rs460105 | rs805303 | rs1047030 | rs1378942 | rs1837164 |
| rs14057 | rs470113 | rs832890 | rs1047891 | rs1401454 | rs1848510 |
| rs20354 | rs479552 | rs839755 | rs1047922 | rs1421811 | rs1859168 |
| rs31864 | rs499425 | rs846963 | rs1048101 | rs1432457 | rs1860157 |
| rs33063 | rs504217 | rs848445 | rs1048238 | rs1438896 | rs1861881 |
| rs35444 | rs504691 | rs849071 | rs1055144 | rs1446468 | rs1870735 |
| rs37060 | rs512083 | rs849074 | rs1060105 | rs1449544 | rs1871190 |
| rs110419 | rs520015 | rs863930 | rs1063281 | rs1458038 | rs1876487 |
| rs134041 | rs521033 | rs867186 | rs1076485 | rs1468520 | rs1878825 |
| rs157678 | rs555754 | rs869396 | rs1077795 | rs1469760 | rs1882289 |
| rs167479 | rs560887 | rs871004 | rs1091811 | rs1475130 | rs1882961 |
| rs177542 | rs574087 | rs871524 | rs1098708 | rs1486236 | rs1886773 |
| rs184457 | rs574835 | rs871606 | rs1100328 | rs1529744 | rs1886914 |
| rs185819 | rs585736 | rs872256 | rs1100329 | rs1530440 | rs1891730 |
| rs187238 | rs587168 | rs873122 | rs1105297 | rs1551355 | rs1906672 |
| rs198846 | rs598682 | rs875106 | rs11110912 | rs1563788 | rs1925153 |
| rs199300 | rs603424 | rs880315 | rs1126464 | rs1563894 | rs1937506 |
| rs200759 | rs606950 | rs882624 | rs1126930 | rs1565716 | rs1947228 |
| rs209405 | rs627425 | rs885582 | rs1133400 | rs1566497 | rs1953126 |
| rs210381 | rs631441 | rs887314 | rs1154214 | rs1585453 | rs1962800 |
| rs1975487 | rs2289081 | rs2761436 | rs3861113 | rs4678915 | rs5810761 |
| rs1982073 | rs2289125 | rs2782980 | rs3889199 | rs4684847 | rs6015450 |
| rs1986971 | rs2289261 | rs2807337 | rs3898618 | rs4699165 | rs6021247 |
| rs1996992 | rs2291435 | rs2808374 | rs3915425 | rs4714224 | rs6031435 |
| rs1997571 | rs2295001 | rs2820037 | rs3918226 | rs4720569 | rs6048704 |
| rs2004776 | rs2298807 | rs2820443 | rs4129585 | rs4728142 | rs6059100 |
| rs2009733 | rs2300481 | rs2854275 | rs4143175 | rs4746172 | rs6060114 |
| rs2014408 | rs2302061 | rs2881492 | rs4245739 | rs4754196 | rs6073142 |
| rs2014912 | rs2303934 | rs2898290 | rs4247374 | rs4757391 | rs6080112 |
| rs2021783 | rs2304130 | rs2920899 | rs4274337 | rs4785955 | rs6081613 |
| rs2024385 | rs2306374 | rs2922895 | rs4286632 | rs4800420 | rs6087449 |
| rs2034618 | rs2316757 | rs2925345 | rs4292285 | rs4810332 | rs6091653 |
| rs2052263 | rs2354862 | rs2929184 | rs4304924 | rs4811601 | rs6095241 |
| rs2065152 | rs2379829 | rs2932538 | rs4342401 | rs4819852 | rs6102925 |
| rs2070744 | rs2384550 | rs2960306 | rs4360494 | rs4823006 | rs6102967 |
| rs2071518 | rs2390258 | rs2969070 | rs4364717 | rs4834735 | rs6108168 |
| rs2104574 | rs2400509 | rs2972146 | rs4373814 | rs4835266 | rs6110544 |
| rs2107595 | rs2404715 | rs2978098 | rs4385883 | rs4841569 | rs6140046 |
| rs2116941 | rs2446849 | rs2978456 | rs4387287 | rs4846049 | rs6141479 |
| rs2138506 | rs2450128 | rs2979470 | rs4411245 | rs4851462 | rs6141767 |
| rs2142141 | rs2467099 | rs3011549 | rs4420291 | rs4873492 | rs6429422 |
| rs2143635 | rs2480171 | rs3097937 | rs4424827 | rs4875958 | rs6434404 |
| rs2162003 | rs2493292 | rs3098186 | rs4443403 | rs4894535 | rs6438857 |
| rs2169137 | rs2498323 | rs3121685 | rs4454254 | rs4896104 | rs6461992 |
| rs2171690 | rs2498586 | rs3135967 | rs4459609 | rs4903064 | rs6487543 |
| rs2175337 | rs2510066 | rs3176336 | rs4475250 | rs4904503 | rs6495122 |
| rs2178452 | rs2513758 | rs3184504 | rs4480845 | rs4908678 | rs6504213 |
| rs2188962 | rs2513877 | rs3191402 | rs4494250 | rs4922591 | rs6511291 |
| rs2189926 | rs2521501 | rs3737801 | rs4499560 | rs4924570 | rs6540125 |
| rs2193635 | rs2579519 | rs3741378 | rs4507125 | rs4925159 | rs6545155 |
| rs2205260 | rs2604972 | rs3742004 | rs4507656 | rs4926499 | rs6551716 |
| rs2222544 | rs2610990 | rs3742182 | rs4530754 | rs4926923 | rs6557876 |
| rs2240736 | rs2613765 | rs3743157 | rs4553000 | rs4952611 | rs6561313 |
| rs2244643 | rs2618647 | rs3745318 | rs4582532 | rs4954192 | rs6565174 |
| rs2246438 | rs2629665 | rs3749594 | rs4590817 | rs4957026 | rs6567160 |
| rs2252865 | rs2631669 | rs3755351 | rs4598218 | rs4965529 | rs6593297 |
| rs2254524 | rs2645466 | rs3771371 | rs4601790 | rs4977492 | rs6595838 |
| rs2267620 | rs2649044 | rs3774372 | rs4631439 | rs4980515 | rs6681713 |
| rs2268062 | rs2656523 | rs3774702 | rs4634143 | rs4980532 | rs6686889 |
| rs2270860 | rs2681492 | rs3783848 | rs4646994 | rs4984496 | rs6712094 |
| rs2277788 | rs2693560 | rs3796822 | rs4651224 | rs5742643 | rs6722745 |
| rs2282123 | rs2707238 | rs3802517 | rs4652875 | rs5744292 | rs6723509 |
| rs2282978 | rs2745599 | rs3820068 | rs4660293 | rs5750482 | rs6728372 |
| rs2285666 | rs2759308 | rs3822857 | rs4664080 | rs5753103 | rs6730325 |
| rs2286615 | rs2760061 | rs3851018 | rs4674114 | rs5767834 | rs6731373 |
| rs6739913 | rs7180952 | rs7914287 | rs9608690 | rs10164193 | rs10913934 |
| rs6749447 | rs7185555 | rs7927515 | rs9609429 | rs10184839 | rs10916082 |
| rs6758859 | rs7187540 | rs7940535 | rs9612192 | rs10189186 | rs10922502 |
| rs6772704 | rs7215084 | rs7945511 | rs9638084 | rs10193543 | rs10923038 |
| rs6774721 | rs7226020 | rs7961152 | rs9662255 | rs10224002 | rs10932679 |
| rs6777317 | rs7235890 | rs7963801 | rs9678851 | rs10233127 | rs10943605 |
| rs6788984 | rs7236548 | rs7965392 | rs9687065 | rs10245696 | rs10948071 |
| rs6797587 | rs7248104 | rs7976167 | rs9693857 | rs10260816 | rs10954174 |
| rs6801957 | rs7250835 | rs7977311 | rs9710247 | rs10267979 | rs10982910 |
| rs6806529 | rs7256564 | rs7977389 | rs9729719 | rs10274928 | rs10988442 |
| rs6823199 | rs7273633 | rs7988232 | rs9810888 | rs10305838 | rs10995311 |
| rs6823767 | rs7297416 | rs8013933 | rs9815354 | rs10407022 | rs10998362 |
| rs6825911 | rs7302981 | rs8014182 | rs9827472 | rs10409243 | rs11008355 |
| rs6861941 | rs7312464 | rs8016306 | rs9833313 | rs10418305 | rs11010905 |
| rs6867399 | rs7313556 | rs8059962 | rs9834975 | rs10427021 | rs11014166 |
| rs6875372 | rs7331680 | rs8068318 | rs9854424 | rs10437954 | rs11021221 |
| rs6878595 | rs7406910 | rs8069739 | rs9854512 | rs10444649 | rs11026586 |
| rs6885719 | rs7437940 | rs8105753 | rs9857362 | rs10460108 | rs11030119 |
| rs6891344 | rs7439567 | rs8111708 | rs9859176 | rs10468291 | rs11031051 |
| rs6911827 | rs7500448 | rs8121443 | rs9860290 | rs10474346 | rs11066280 |
| rs6925750 | rs7514579 | rs8125763 | rs9865843 | rs10520555 | rs11067763 |
| rs6941056 | rs7515635 | rs9302885 | rs9875380 | rs10732433 | rs11080134 |
| rs6959688 | rs7519279 | rs9306160 | rs9885577 | rs10743086 | rs11085020 |
| rs6963105 | rs7524019 | rs9316206 | rs9885632 | rs10747570 | rs11087740 |
| rs6966942 | rs7555285 | rs9323988 | rs9886665 | rs10760117 | rs11091046 |
| rs6969780 | rs7591091 | rs9329123 | rs9888615 | rs10761530 | rs11108209 |
| rs6996733 | rs7592578 | rs9329124 | rs9899540 | rs10766533 | rs11112548 |
| rs6997709 | rs7599598 | rs9329125 | rs9904409 | rs10770612 | rs11128722 |
| rs7009170 | rs7606205 | rs9337951 | rs9932220 | rs10779936 | rs11139596 |
| rs7020564 | rs7608483 | rs9349379 | rs9932866 | rs10782230 | rs11141731 |
| rs7023828 | rs7611674 | rs9368222 | rs9976596 | rs10787517 | rs11145807 |
| rs7043304 | rs7630745 | rs9372498 | rs10008637 | rs10818775 | rs11154027 |
| rs7045409 | rs7694000 | rs9392172 | rs10025123 | rs10823136 | rs11159091 |
| rs7048294 | rs7694643 | rs9401090 | rs10048404 | rs10826995 | rs11160085 |
| rs7090758 | rs7710854 | rs9431431 | rs10050665 | rs10830963 | rs11168245 |
| rs7103648 | rs7719781 | rs9449350 | rs10057188 | rs10842991 | rs11187142 |
| rs7118863 | rs7734819 | rs9456648 | rs10059921 | rs10850411 | rs11191156 |
| rs7126805 | rs7753695 | rs9472135 | rs10062049 | rs10850519 | rs11191548 |
| rs7129220 | rs7763294 | rs9479509 | rs10065231 | rs10858966 | rs11195417 |
| rs7132012 | rs7765526 | rs9486916 | rs10069690 | rs10860812 | rs11195419 |
| rs7134060 | rs7799039 | rs9526707 | rs10077885 | rs10864859 | rs11197813 |
| rs7137749 | rs7845722 | rs9532243 | rs10078021 | rs10873612 | rs11210029 |
| rs7144602 | rs7861040 | rs9549297 | rs10087782 | rs10887914 | rs11222084 |
| rs7157104 | rs7869756 | rs9549328 | rs10107145 | rs10889553 | rs11222386 |
| rs7178615 | rs7912283 | rs9563529 | rs10149796 | rs10906391 | rs11229457 |
| rs11231757 | rs11701512 | rs12405515 | rs13107325 | rs17428471 | rs35199222 |
| rs11237967 | rs11711623 | rs12408022 | rs13112725 | rs17454517 | rs35213536 |
| rs11237968 | rs11716531 | rs12434998 | rs13122790 | rs17477177 | rs35261357 |
| rs11237969 | rs11725861 | rs12454712 | rs13139571 | rs17516329 | rs35287509 |
| rs11237970 | rs11730129 | rs12474050 | rs13149209 | rs17608766 | rs35410524 |
| rs11237971 | rs11740074 | rs12486605 | rs13179413 | rs17638167 | rs35450617 |
| rs11248862 | rs11746388 | rs12506214 | rs13192976 | rs17643123 | rs35479618 |
| rs11249785 | rs11749194 | rs12511987 | rs13205180 | rs17857342 | rs35504735 |
| rs11442819 | rs11760407 | rs12515422 | rs13209747 | rs17880989 | rs35529250 |
| rs11465670 | rs11770630 | rs12515541 | rs13238550 | rs28362590 | rs35590893 |
| rs11466111 | rs11771693 | rs12521868 | rs13240040 | rs28377357 | rs35696236 |
| rs11537751 | rs11789875 | rs12574332 | rs13253358 | rs28408355 | rs35783704 |
| rs11542299 | rs11853359 | rs12579720 | rs13290326 | rs28470843 | rs35796750 |
| rs11556924 | rs11876341 | rs12583637 | rs13333226 | rs28498002 | rs35981664 |
| rs11564022 | rs11901929 | rs12596053 | rs13359291 | rs28499085 | rs36006409 |
| rs11571376 | rs11909120 | rs12605156 | rs13403122 | rs28558491 | rs36010659 |
| rs11579440 | rs11923667 | rs12606620 | rs13407401 | rs28558845 | rs36022378 |
| rs11585169 | rs11930293 | rs12627651 | rs13420463 | rs28578714 | rs36047283 |
| rs11592107 | rs11932929 | rs12628032 | rs16823124 | rs28590346 | rs36083386 |
| rs11601860 | rs11938045 | rs12638085 | rs16833934 | rs28611491 | rs41457144 |
| rs11607056 | rs11941467 | rs12652175 | rs16849225 | rs28621435 | rs41475048 |
| rs11615689 | rs11953630 | rs12668436 | rs16851397 | rs28633979 | rs45474499 |
| rs11621835 | rs11977526 | rs12694277 | rs16870400 | rs28667801 | rs55641580 |
| rs11623535 | rs11991469 | rs12703989 | rs16948048 | rs28669318 | rs55684003 |
| rs11626434 | rs12034319 | rs12705090 | rs16998073 | rs28675079 | rs55701159 |
| rs11627326 | rs12042924 | rs12731740 | rs17004869 | rs33966350 | rs55732192 |
| rs11628933 | rs12050260 | rs12744757 | rs17030613 | rs33996239 | rs55747751 |
| rs11629850 | rs12052761 | rs12797948 | rs17035181 | rs34022638 | rs55750340 |
| rs11630824 | rs12052878 | rs12799484 | rs17059668 | rs34072724 | rs55780018 |
| rs11631967 | rs12078697 | rs12807220 | rs17074589 | rs34130368 | rs55829085 |
| rs11632112 | rs12118102 | rs12884621 | rs17074592 | rs34161718 | rs55891215 |
| rs11632436 | rs12129649 | rs12906962 | rs17074601 | rs34163044 | rs55935819 |
| rs11634028 | rs12142296 | rs12921187 | rs17080102 | rs34324971 | rs56013986 |
| rs11634676 | rs12146487 | rs12938803 | rs17097182 | rs34413141 | rs56118860 |
| rs11634851 | rs12153395 | rs12940887 | rs17115100 | rs34430710 | rs56233017 |
| rs11639856 | rs12172847 | rs12941318 | rs17115145 | rs34517439 | rs56236159 |
| rs11642631 | rs12184466 | rs12946454 | rs17119370 | rs34570306 | rs56249585 |
| rs11643209 | rs12195276 | rs12958173 | rs17224476 | rs34584627 | rs56345595 |
| rs11646213 | rs12216497 | rs12983238 | rs17248480 | rs34591516 | rs56352451 |
| rs11665020 | rs12216886 | rs12990959 | rs17249754 | rs34705210 | rs56844452 |
| rs11681462 | rs12325702 | rs13001283 | rs17287293 | rs34783010 | rs57327054 |
| rs11689667 | rs12360999 | rs13002573 | rs17321041 | rs34865359 | rs57786342 |
| rs11690961 | rs12362593 | rs13014371 | rs17339050 | rs34872471 | rs57927100 |
| rs11694601 | rs12363915 | rs13042148 | rs17367504 | rs34941092 | rs59333122 |
| rs11701033 | rs12374077 | rs13082711 | rs17396055 | rs34983854 | rs59986178 |
| rs60148403 | rs62158170 | rs72659998 | rs73006725 | rs75905900 | rs79211524 |
| rs60191654 | rs62167177 | rs72683923 | rs73006729 | rs75961402 | rs79409628 |
| rs60199046 | rs62169544 | rs72688070 | rs73006745 | rs76164690 | rs79523138 |
| rs60255247 | rs62229372 | rs72704264 | rs73033340 | rs76217164 | rs79598313 |
| rs60761033 | rs62270945 | rs72719160 | rs73046792 | rs76326501 | rs79917357 |
| rs61040371 | rs62278541 | rs72761109 | rs73049928 | rs76452347 | rs79960663 |
| rs61272231 | rs62373688 | rs72765298 | rs73080726 | rs76627118 | rs80202922 |
| rs61448762 | rs62380354 | rs72795925 | rs73099903 | rs76719272 | rs80346118 |
| rs61653296 | rs62491354 | rs72799341 | rs73105827 | rs76785029 | rs111245230 |
| rs61735998 | rs62503324 | rs72812846 | rs73158427 | rs76987554 | rs111777102 |
| rs61760904 | rs62523863 | rs72816333 | rs73161324 | rs77279095 | rs111959824 |
| rs61767086 | rs62524579 | rs72834453 | rs73171158 | rs77413490 | rs112184198 |
| rs61886886 | rs62526122 | rs72842207 | rs73187288 | rs77573976 | rs112260610 |
| rs61892344 | rs63418562 | rs72844590 | rs73727605 | rs77692990 | rs112280096 |
| rs61912333 | rs66774912 | rs72847884 | rs73802496 | rs78049276 | rs112557609 |
| rs61926181 | rs66887589 | rs72847885 | rs74181299 | rs78151625 | rs113134141 |
| rs62004794 | rs66990951 | rs72851229 | rs74774746 | rs78378222 | rs113866309 |
| rs62011052 | rs67330701 | rs72876037 | rs75305034 | rs78474310 | rs114275780 |
| rs62012628 | rs67720684 | rs72914576 | rs75389451 | rs78648104 | rs114503346 |
| rs62053102 | rs67976715 | rs72930904 | rs75507123 | rs78998485 | rs115172170 |
| rs62080325 | rs71543920 | rs73006721 | rs75666605 | rs79089478 | rs115231027 |
| rs62104477 | rs72613227 | rs73006723 | rs75902664 | rs79146658 | rs115245297 |
| rs115795127 | rs139184666 | rs142449193 | rs147696085 | rs190194639 | rs551011992 |
| rs117006983 | rs139236208 | rs142892876 | rs150194832 | rs191784289 | rs568998724 |
| rs117206641 | rs139354822 | rs143112823 | rs150266910 | rs200303279 | rs569158324 |
| rs117233107 | rs139385870 | rs143118162 | rs150816167 | rs200337503 | rs1000514384 |
| rs117539635 | rs139703184 | rs143167197 | rs151054210 | rs200999181 | rs1165104995 |
| rs138285687 | rs141216986 | rs144073138 | rs151168737 | rs528650186 | rs1229536170 |
| rs138643143 | rs141767645 | rs147212971 | rs187680191 | rs528908640 | rs1299013576 |
| rs138877676 | rs141979279 | rs147216662 | rs189267552 | rs547122124 | rs1441067441 |
|  |  |  |  |  | rs1772315630 |
