# Supplementary material for: Polygenic risk scores predict blood pressure changes independent of dietary intervention: a secondary analysis of the NUPRESS trial
Source: Eur J Nutr. 2026 Aug 1;65(5):225. doi: 10.1007/s00394-026-04072-x (PMC13428793; doi:10.1007/s00394-026-04072-x)
Supplement: Supplementary file 3 — Supplementary Material 3 [file 394_2026_4072_MOESM3_ESM.docx]

**Article title:** Polygenic risk scores predict blood pressure changes independent of dietary intervention: a secondary analysis of the NUPRESS trial

**Journal name:** European Journal of Nutrition

**Authors:** Luciana C Holzbach^a,b^, Aline Marcadenti^c,d,e^, Angela C Bersch-Fereira^f^, Rachel H Vieira Machado^c^, Ana Paula P F Carvalho^g^, Sônia L Pinto^1^, Andreza M Penafort^h^, Alexandre S G Coelho^i^, Cristiane Cominetti^b*^

***Corresponding author:** Cristiane Cominetti. Nutritional Genomics Research Group, School of Nutrition. Federal University of Goiás. Rua 227, s/n, Quadra 68, Leste Universitário, CEP 74605080, Goiânia, GO, Brazil. Phone: +55-62-32096270 ext. 210. Fax: + 55-62-32096273. e-mail: [ccominetti@ufg.br](mailto:ccominetti@ufg.br)

**Table S2** Selected SNPs and their effects for constructing the ΔSBP and ΔDBP polygenic risk scores.

| **ΔSBP** | | | | | | | | | |
| --- | --- | --- | --- | --- | --- | --- | --- | --- | --- |
| **SNP** | **Prob_ID** | **Cro.** | **Position** | **Ref. allele** | **Alt. allele** | **ꞵ** | **r^2^** | **p-value** |  |
| rs12731740 | AX-11225162 | 1 | 207851475 | C | T | -8.64 | 0.03 | 0.01 |  |
| rs1967017 | AX-11356525 | 1 | 145711421 | A | G | -3.49 | 0.01 | 0.06 |  |
| rs9436302 | AX-13070742 | 1 | 65430878 | G | A | 3.62 | 0.01 | 0.06 |  |
| rs12120171 | AX-16890840 | 1 | 221184625 | G | A | 3.93 | 0.01 | 0.05 |  |
| rs2072929 | AX-30213431 | 1 | 1755504 | T | C | -3.10 | 0.01 | 0.09 |  |
| rs34517439 | AX-32353673 | 1 | 77984833 | C | A | 9.47 | 0.03 | 0.01 |  |
| rs114455337 | AX-37386831 | 1 | 56543185 | C | T | -9.92 | 0.01 | 0.05 |  |
| rs4660293 | AX-39708829 | 1 | 39562508 | A | G | 4.02 | 0.01 | 0.08 |  |
| rs1801133 | AX-51283185 | 1 | 11796321 | G | A | -3.80 | 0.01 | 0.06 |  |
| rs4671696 | AX-11516827 | 2 | 65876852 | A | G | 8.76 | 0.02 | 0.02 |  |
| rs79244098 | AX-13775842 | 2 | 182358343 | T | C | 7.75 | 0.02 | 0.01 |  |
| rs4952611 | AX-13944854 | 2 | 40340603 | C | T | -3.33 | 0.01 | 0.08 |  |
| rs6730957 | AX-33393487 | 2 | 171524363 | C | A | 4.79 | 0.03 | 0.02 |  |
| rs13426587 | AX-40774151 | 2 | 177125654 | T | C | -7.51 | 0.02 | 0.04 |  |
| rs9306895 | AX-40819159 | 2 | 20678393 | T | C | 3.79 | 0.01 | 0.06 |  |
| rs10932679 | AX-40837949 | 2 | 216787868 | A | T | -4.39 | 0.01 | 0.07 |  |
| rs2356976 | AX-41057649 | 2 | 9154059 | C | T | -4.59 | 0.02 | 0.03 |  |
| rs2176502 | AX-57625317 | 2 | 188786502 | G | T | -9.11 | 0.01 | 0.09 |  |
| rs9831772 | AX-11704801 | 3 | 193648150 | T | G | 8.64 | 0.00 | 0.02 |  |
| rs9843455 | AX-14326838 | 3 | 56204378 | G | T | -12.42 | 0.02 | 0.02 |  |
| rs76731918 | AX-14366994 | 3 | 73210540 | G | A | -5.70 | 0.01 | 0.05 |  |
| rs903063 | AX-34104263 | 3 | 14916456 | C | T | -5.54 | 0.01 | 0.07 |  |
| rs62296619 | AX-34183481 | 3 | 177209731 | C | T | -6.39 | 0.03 | 0.01 |  |
| rs11130158 | AX-34337253 | 3 | 48156810 | C | A | 4.74 | 0.03 | 0.01 |  |
| rs36022378 | AX-34340453 | 3 | 49876272 | T | C | 4.25 | 0.01 | 0.08 |  |
| rs743757 | AX-41246945 | 3 | 50438947 | G | C | 3.14 | 0.01 | 0.09 |  |
| rs9835280 | AX-41279451 | 3 | 70874810 | G | A | 9.72 | 0.02 | 0.02 |  |
| rs10103 | AX-14461809 | 4 | 119035530 | C | T | 4.90 | 0.01 | 0.08 |  |
| rs4690016 | AX-14644249 | 4 | 2705405 | G | A | 3.94 | 0.02 | 0.02 |  |
| rs1347189 | AX-41348663 | 4 | 123881748 | G | A | 3.68 | 0.02 | 0.04 |  |
| *** | AX-150231835 | 4 | 108092589 | G | A | -12.95 | 0.02 | 0.00 |  |
| rs1801058 | AX-165876720 | 4 | 3037423 | T | C | 3.74 | 0.01 | 0.08 |  |
| rs2898029 | AX-11425691 | 5 | 57804261 | C | A | 5.93 | 0.01 | 0.09 |  |
| rs31864 | AX-12549706 | 5 | 158793185 | G | A | -3.58 | 0.02 | 0.04 |  |
| rs72792276 | AX-14886486 | 5 | 128028434 | C | A | -7.36 | 0.03 | 0.00 |  |
| rs246974 | AX-15100401 | 5 | 68710860 | C | T | 3.83 | 0.01 | 0.07 |  |
| rs10479613 | AX-35235083 | 5 | 177972563 | G | A | 7.82 | 0.02 | 0.03 |  |
| rs79857689 | AX-38254501 | 5 | 156722682 | C | T | -10.36 | 0.01 | 0.06 |  |
| rs17119056 | AX-41616893 | 5 | 140695889 | C | T | -5.85 | 0.01 | 0.08 |  |
| rs409558 | AX-11490559 | 6 | 31740370 | T | C | -3.93 | 0.01 | 0.05 |  |
| rs5370 | AX-11543724 | 6 | 12296022 | G | T | 4.03 | 0.01 | 0.09 |  |
| rs11154027 | AX-15217246 | 6 | 121460244 | T | C | -3.30 | 0.01 | 0.06 |  |
| rs7763581 | AX-35623897 | 6 | 1614132 | T | G | 4.22 | 0.02 | 0.02 |  |
| rs7757881 | AX-35642407 | 6 | 161290329 | A | G | 3.34 | 0.01 | 0.07 |  |
| rs35651100 | AX-35907109 | 6 | 83596999 | G | T | 5.18 | 0.02 | 0.04 |  |
| rs17080102 | AX-41881911 | 6 | 150683634 | G | C | 4.34 | 0.01 | 0.09 |  |
| rs13205180 | AX-41994905 | 6 | 51967696 | C | T | 3.68 | 0.01 | 0.06 |  |
| *** | AX-12579180 | 7 | 47014233 | T | C | 16.4 | 0.05 | 0.00 |  |
| rs17477177 | AX-15512704 | 7 | 106771412 | T | C | -5.65 | 0.03 | 0.01 |  |
| rs1800795 | AX-15644586 | 7 | 22727026 | C | G | 5.42 | 0.03 | 0.01 |  |
| rs4723948 | AX-15696604 | 7 | 4629505 | T | C | 3.29 | 0.01 | 0.08 |  |
| rs10225543 | AX-36117481 | 7 | 156615953 | C | G | -4.49 | 0.02 | 0.03 |  |
| rs1048101 | AX-11108881 | 8 | 26770511 | A | G | 3.32 | 0.01 | 0.05 |  |
| rs2100347 | AX-11367250 | 8 | 63587046 | C | T | 3.90 | 0.02 | 0.05 |  |
| rs56046428 | AX-15862728 | 8 | 128471034 | G | A | -6.29 | 0.01 | 0.06 |  |
| rs16879552 | AX-15945003 | 8 | 32553698 | C | T | -5.97 | 0.01 | 0.05 |  |
| rs4129585 | AX-42358801 | 8 | 142231572 | A | C | 4.29 | 0.02 | 0.03 |  |
| rs5894304 | AX-107806765 | 8 | 115941418 | AG | - | 3.69 | 0.02 | 0.00 |  |
| rs3834373 | AX-121335847 | 8 | 22563446 | - | G | 4.63 | 0.02 | 0.04 |  |
| rs66698579 | AX-148434654 | 8 | 142980160 | G | A | -4.98 | 0.01 | 0.07 |  |
| rs866746113 | AX-151385032 | 8 | 80483076 | CTT | - | -9.43 | 0.01 | 0.05 |  |
| rs644383 | AX-42584861 | 9 | 208665 | A | G | -3.85 | 0.02 | 0.02 |  |
| rs11444574 | AX-151163429 | 9 | 127546951 | - | A | -5.07 | 0.00 | 0.04 |  |
| rs74629554 | AX-16462865 | 11 | 102210664 | A | G | 14.04 | 0.03 | 0.01 |  |
| rs80040482 | AX-16521768 | 11 | 130909515 | C | A | -10.62 | 0.01 | 0.07 |  |
| rs58320648 | AX-16642616 | 11 | 58440427 | G | A | -10.49 | 0.02 | 0.04 |  |
| rs74053139 | AX-16740821 | 11 | 8746201 | G | A | -6.75 | 0.01 | 0.06 |  |
| rs34493990 | AX-29869741 | 11 | 116225050 | G | T | 17.02 | 0.03 | 0.01 |  |
| *** | AX-29984991 | 11 | 1995432 | T | C | -4.04 | 0.02 | 0.04 |  |
| rs67330701 | AX-30222009 | 11 | 69312240 | C | T | 6.87 | 0.01 | 0.07 |  |
| rs11236204 | AX-30257241 | 11 | 74670674 | G | A | -6.56 | 0.04 | 0.00 |  |
| rs11227229 | AX-39137523 | 11 | 65586679 | G | A | -4.52 | 0.03 | 0.01 |  |
| rs1946518 | AX-165887391 | 11 | 112164735 | T | G | 5.84 | 0.04 | 0.00 |  |
| rs2384550 | AX-11388634 | 12 | 114914926 | G | A | -4.77 | 0.03 | 0.01 |  |
| rs75661051 | AX-30527363 | 12 | 122114473 | A | G | -5.19 | 0.02 | 0.02 |  |
| rs2728586 | AX-30620631 | 12 | 20022256 | T | C | 3.97 | 0.01 | 0.06 |  |
| *** | AX-30624013 | 12 | 20603868 | A | G | -9.21 | 0.01 | 0.06 |  |
| rs7309896 | AX-39453949 | 12 | 50381673 | C | G | 9.69 | 0.01 | 0.06 |  |
| rs11618348 | AX-11162875 | 13 | 58041112 | A | C | 3.25 | 0.01 | 0.07 |  |
| rs6561314 | AX-11572123 | 13 | 46524907 | C | T | -9.20 | 0.04 | 0.00 |  |
| rs56125130 | AX-17238513 | 13 | 77887647 | G | A | -10.87 | 0.02 | 0.04 |  |
| rs3742182 | AX-30993773 | 13 | 110722785 | C | T | -4.94 | 0.01 | 0.08 |  |
| rs9549328 | AX-31004697 | 13 | 112981842 | C | T | -5.47 | 0.03 | 0.01 |  |
| rs9548378 | AX-31062351 | 13 | 31617720 | G | T | -5.59 | 0.01 | 0.07 |  |
| rs649363 | AX-39577505 | 13 | 20985148 | A | G | 5.49 | 0.03 | 0.01 |  |
| rs6575059 | AX-39835319 | 14 | 89386550 | G | A | 6.22 | 0.04 | 0.00 |  |
| rs10851885 | AX-11126951 | 15 | 76012162 | A | G | 4.14 | 0.01 | 0.05 |  |
| rs10906982 | AX-11131010 | 15 | 83899406 | T | A | 4.66 | 0.02 | 0.02 |  |
| rs16974951 | AX-12482730 | 15 | 84618041 | C | T | -5.24 | 0.01 | 0.08 |  |
| rs11634851 | AX-12973951 | 15 | 80736624 | C | G | 3.92 | 0.02 | 0.03 |  |
| rs7174546 | AX-13004604 | 15 | 96094340 | A | G | 4.01 | 0.01 | 0.06 |  |
| rs75023556 | AX-31718593 | 15 | 78859743 | C | T | -4.72 | 0.01 | 0.06 |  |
| rs1378942 | AX-39946899 | 15 | 74785026 | C | A | -3.21 | 0.01 | 0.08 |  |
| rs2759308 | AX-39956643 | 15 | 80723886 | G | A | -3.66 | 0.02 | 0.04 |  |
| rs3964074 | AX-13104436 | 16 | 74133092 | T | C | 3.63 | 0.01 | 0.05 |  |
| rs117721843 | AX-31890609 | 16 | 4248428 | G | A | 9.26 | 0.02 | 0.05 |  |
| rs74029173 | AX-32030429 | 16 | 81542157 | G | C | 7.52 | 0.01 | 0.09 |  |
| rs10782001 | AX-40026155 | 16 | 30931304 | G | A | -4.73 | 0.03 | 0.01 |  |
| rs9925842 | AX-40070485 | 16 | 66881159 | G | A | 3.86 | 0.01 | 0.06 |  |
| rs4308 | AX-32261395 | 17 | 63482264 | A | G | -4.36 | 0.02 | 0.03 |  |
| rs17881556 | AX-32331559 | 17 | 7667551 | T | C | 10.34 | 0.03 | 0.01 |  |
| rs116970340 | AX-37793699 | 17 | 42559750 | A | G | -7.15 | 0.02 | 0.04 |  |
| rs5415 | AX-40237521 | 17 | 7281162 | T | C | -5.23 | 0.02 | 0.02 |  |
| rs10852766 | AX-40237881 | 17 | 75955783 | T | C | 3.92 | 0.02 | 0.03 |  |
| rs12962859 | AX-11234036 | 18 | 62553424 | A | G | -5.30 | 0.02 | 0.01 |  |
| rs6566073 | AX-13393772 | 18 | 75321417 | T | C | 3.97 | 0.02 | 0.03 |  |
| rs62092240 | AX-32484365 | 18 | 50752421 | T | C | -5.42 | 0.02 | 0.04 |  |
| *** | AX-37823189 | 18 | 33578991 | C | T | -6.20 | 0.01 | 0.09 |  |
| rs10425864 | AX-32816617 | 19 | 7256536 | A | G | -5.14 | 0.02 | 0.02 |  |
| rs79386654 | AX-32816651 | 19 | 7260800 | A | G | 9.62 | 0.01 | 0.05 |  |
| rs1800471 | AX-112180963 | 19 | 41352971 | C | G | -6.46 | 0.01 | 0.07 |  |
| rs12627651 | AX-40719701 | 21 | 43340723 | G | A | 3.66 | 0.01 | 0.07 |  |
| rs17730978 | AX-33595265 | 22 | 30358377 | T | C | 8.73 | 0.02 | 0.03 |  |
| rs6008308 | AX-40942267 | 22 | 47615353 | A | C | -3.28 | 0.01 | 0.07 |  |
| **ΔDBP** | | | | | | | | | |
| **SNP** | **Prob_ID** | **Cro.** | **Position** | **Ref. allele** | **Alt. allele** | **ꞵ** | **r^2^** | **p-value** |  |
| rs17382975 | AX-12874460 | 1 | 50543518 | A | G | -4.16 | 0.01 | 0.10 |  |
| rs9436302 | AX-13070742 | 1 | 65430878 | G | A | 3.85 | 0.03 | 0.01 |  |
| rs12120171 | AX-16890840 | 1 | 221184625 | G | A | 3.12 | 0.02 | 0.03 |  |
| rs114455337 | AX-37386831 | 1 | 56543185 | C | T | -7.20 | 0.01 | 0.05 |  |
| rs7535237 | AX-40075485 | 1 | 66604305 | C | T | -5.71 | 0.02 | 0.04 |  |
| rs2252865 | AX-40304475 | 1 | 8362616 | T | C | -3.42 | 0.02 | 0.02 |  |
| rs41267642 | AX-151592102 | 1 | 180884952 | C | G | 8.49 | 0.02 | 0.03 |  |
| rs1567229 | AX-11276297 | 2 | 72879465 | C | T | 3.51 | 0.01 | 0.07 |  |
| rs2903704 | AX-11426173 | 2 | 55578304 | T | C | -8.08 | 0.01 | 0.05 |  |
| rs75928112 | AX-13716842 | 2 | 152762987 | A | G | 7.15 | 0.01 | 0.06 |  |
| rs16849225 | AX-13738302 | 2 | 164050310 | C | T | 2.93 | 0.01 | 0.08 |  |
| rs79244098 | AX-13775842 | 2 | 182358343 | T | C | 6.10 | 0.03 | 0.01 |  |
| rs71435601 | AX-13831171 | 2 | 21205417 | A | G | -3.98 | 0.01 | 0.08 |  |
| rs2540949 | AX-33875815 | 2 | 65057097 | A | T | -3.58 | 0.03 | 0.01 |  |
| rs13426587 | AX-40774151 | 2 | 177125654 | T | C | -6.57 | 0.03 | 0.01 |  |
| rs1438053 | AX-40775487 | 2 | 177854941 | A | G | 3.76 | 0.02 | 0.01 |  |
| rs2176502 | AX-57625317 | 2 | 188786502 | G | T | -8.59 | 0.02 | 0.02 |  |
| rs7608623 | AX-96087565 | 2 | 23726083 | G | T | -2.88 | 0.02 | 0.04 |  |
| rs4607103 | AX-11513383 | 3 | 64726228 | C | T | 3.20 | 0.02 | 0.02 |  |
| rs62296619 | AX-34183481 | 3 | 177209731 | C | T | -3.00 | 0.01 | 0.09 |  |
| rs9311612 | AX-34359881 | 3 | 56679947 | G | A | 3.86 | 0.02 | 0.04 |  |
| *** | AX-38045147 | 3 | 30363047 | T | C | -6.27 | 0.01 | 0.09 |  |
| rs4684160 | AX-41120103 | 3 | 13786724 | G | C | 2.67 | 0.01 | 0.07 |  |
| rs743757 | AX-41246945 | 3 | 50438947 | G | C | 2.44 | 0.01 | 0.07 |  |
| rs1487617 | AX-11268205 | 4 | 38388208 | A | G | -2.26 | 0.01 | 0.09 |  |
| rs4835265 | AX-14520316 | 4 | 145900258 | C | A | -3.97 | 0.02 | 0.02 |  |
| rs74960468 | AX-14574742 | 4 | 168794764 | G | A | 6.32 | 0.02 | 0.04 |  |
| rs11132173 | AX-14610600 | 4 | 183280756 | G | A | 2.68 | 0.02 | 0.00 |  |
| rs62357979 | AX-14610658 | 4 | 183304568 | C | T | 4.07 | 0.03 | 0.01 |  |
| rs4690016 | AX-14644249 | 4 | 2705405 | G | A | 3.88 | 0.04 | 0.00 |  |
| rs13146355 | AX-14760177 | 4 | 76490987 | G | A | 3.55 | 0.02 | 0.01 |  |
| rs10003916 | AX-34723883 | 4 | 183295933 | A | G | -2.40 | 0.01 | 0.08 |  |
| rs13111200 | AX-34961513 | 4 | 83004451 | C | T | 3.31 | 0.01 | 0.05 |  |
| rs1347189 | AX-41348663 | 4 | 123881748 | G | A | 3.39 | 0.03 | 0.01 |  |
| rs12499599 | AX-41467457 | 4 | 36100140 | C | T | -3.68 | 0.02 | 0.00 |  |
| rs17148568 | AX-11310294 | 5 | 121854755 | A | C | -3.21 | 0.01 | 0.10 |  |
| rs2898029 | AX-11425691 | 5 | 57804261 | C | A | 5.33 | 0.02 | 0.03 |  |
| rs80213949 | AX-15073298 | 5 | 56573795 | C | A | -6.20 | 0.02 | 0.00 |  |
| rs246974 | AX-15100401 | 5 | 68710860 | C | T | 2.90 | 0.01 | 0.06 |  |
| rs79857689 | AX-38254501 | 5 | 156722682 | C | T | -7.57 | 0.01 | 0.06 |  |
| rs34693 | AX-41757605 | 5 | 66992913 | T | G | -3.57 | 0.03 | 0.01 |  |
| rs5370 | AX-11543724 | 6 | 12296022 | G | T | 3.23 | 0.01 | 0.06 |  |
| rs16884761 | AX-12476519 | 6 | 54131703 | G | T | -4.53 | 0.02 | 0.04 |  |
| rs3822857 | AX-15203838 | 6 | 115992768 | G | C | 2.56 | 0.01 | 0.06 |  |
| rs449789 | AX-15300924 | 6 | 159278093 | C | G | -3.49 | 0.01 | 0.05 |  |
| rs7763581 | AX-35623897 | 6 | 1614132 | T | G | 2.42 | 0.01 | 0.07 |  |
| rs9398064 | AX-35939239 | 6 | 96452774 | C | A | 6.20 | 0.03 | 0.01 |  |
| rs62426321 | AX-67462900 | 6 | 126809757 | C | T | -7.89 | 0.02 | 0.04 |  |
| rs6459694 | AX-11565496 | 7 | 156621027 | A | G | -2.56 | 0.00 | 0.08 |  |
| rs1055144 | AX-12393686 | 7 | 25831489 | C | T | -3.27 | 0.02 | 0.08 |  |
| *** | AX-12579180 | 7 | 47014233 | T | C | 10.61 | 0.03 | 0.01 |  |
| rs1800795 | AX-15644586 | 7 | 22727026 | C | G | 3.41 | 0.02 | 0.02 |  |
| rs4723948 | AX-15696604 | 7 | 4629505 | T | C | 2.37 | 0.01 | 0.08 |  |
| rs12697978 | AX-36116943 | 7 | 156516948 | T | C | -2.63 | 0.01 | 0.09 |  |
| rs1860157 | AX-42154833 | 7 | 156634507 | C | A | 4.23 | 0.02 | 0.02 |  |
| rs2100347 | AX-11367250 | 8 | 63587046 | C | T | 2.76 | 0.01 | 0.06 |  |
| rs7820237 | AX-36401541 | 8 | 10789007 | T | C | 2.82 | 0.02 | 0.04 |  |
| rs4129585 | AX-42358801 | 8 | 142231572 | A | C | 4.78 | 0.05 | 0.00 |  |
| rs10121001 | AX-11094156 | 9 | 82514520 | T | G | -2.74 | 0.01 | 0.05 |  |
| rs35287509 | AX-11461397 | 9 | 10594635 | T | C | -2.69 | 0.01 | 0.06 |  |
| rs77027750 | AX-37012691 | 9 | 4335651 | A | G | -7.86 | 0.02 | 0.01 |  |
| rs10994431 | AX-11136933 | 10 | 60617674 | T | G | -3.37 | 0.01 | 0.08 |  |
| rs115177963 | AX-37503271 | 10 | 125023588 | T | C | 4.69 | 0.01 | 0.07 |  |
| rs11237964 | AX-11152813 | 11 | 79677112 | A | G | 4.25 | 0.02 | 0.03 |  |
| rs74629554 | AX-16462865 | 11 | 102210664 | A | G | 10.43 | 0.03 | 0.01 |  |
| rs58320648 | AX-16642616 | 11 | 58440427 | G | A | -8.92 | 0.02 | 0.01 |  |
| rs11236204 | AX-30257241 | 11 | 74670674 | G | A | -4.36 | 0.04 | 0.01 |  |
| rs1946518 | AX-165887391 | 11 | 112164735 | T | G | 3.83 | 0.03 | 0.01 |  |
| rs2384550 | AX-11388634 | 12 | 114914926 | G | A | -4.61 | 0.05 | 0.00 |  |
| rs35444 | AX-39295911 | 12 | 115114632 | A | G | 3.02 | 0.02 | 0.03 |  |
| rs10850519 | AX-39296633 | 12 | 115490635 | G | C | 2.68 | 0.01 | 0.06 |  |
| rs6561314 | AX-11572123 | 13 | 46524907 | C | T | -5.63 | 0.02 | 0.02 |  |
| rs9549328 | AX-31004697 | 13 | 112981842 | C | T | -2.85 | 0.01 | 0.08 |  |
| rs8016306 | AX-12805892 | 14 | 63461828 | A | G | -2.34 | 0.01 | 0.08 |  |
| rs2146103 | AX-12873286 | 14 | 100273892 | C | A | 2.74 | 0.01 | 0.08 |  |
| rs11160652 | AX-39715921 | 14 | 101532327 | G | A | 2.50 | 0.01 | 0.06 |  |
| rs6575059 | AX-39835319 | 14 | 89386550 | G | A | 2.88 | 0.01 | 0.06 |  |
| rs10851885 | AX-11126951 | 15 | 76012162 | A | G | 2.92 | 0.01 | 0.06 |  |
| rs10906982 | AX-11131010 | 15 | 83899406 | T | A | 3.94 | 0.03 | 0.01 |  |
| rs7169375 | AX-12908192 | 15 | 41018848 | T | C | 2.70 | 0.02 | 0.04 |  |
| rs11634851 | AX-12973951 | 15 | 80736624 | C | G | 2.85 | 0.02 | 0.03 |  |
| rs2759308 | AX-39956643 | 15 | 80723886 | G | A | -2.61 | 0.02 | 0.05 |  |
| rs6497755 | AX-11568302 | 16 | 24775874 | C | A | -2.86 | 0.02 | 0.03 |  |
| rs7499352 | AX-11626357 | 16 | 6843365 | C | G | 3.21 | 0.01 | 0.05 |  |
| rs3964074 | AX-13104436 | 16 | 74133092 | T | C | 3.10 | 0.02 | 0.02 |  |
| rs59333122 | AX-31839079 | 16 | 19138674 | C | A | 2.74 | 0.02 | 0.04 |  |
| rs117046385 | AX-31972551 | 16 | 69104022 | G | A | -8.47 | 0.03 | 0.01 |  |
| rs10782001 | AX-40026155 | 16 | 30931304 | G | A | -2.38 | 0.01 | 0.07 |  |
| rs11871159 | AX-12421619 | 17 | 46134039 | T | C | -9.57 | 0.03 | 0.01 |  |
| rs9896752 | AX-12671218 | 17 | 45852834 | T | C | -3.98 | 0.01 | 0.06 |  |
| rs4308 | AX-32261395 | 17 | 63482264 | A | G | -2.73 | 0.01 | 0.07 |  |
| rs116970340 | AX-37793699 | 17 | 42559750 | A | G | -4.86 | 0.01 | 0.06 |  |
| rs2465429 | AX-40213463 | 17 | 62689122 | A | G | 3.76 | 0.01 | 0.05 |  |
| rs7236548 | AX-12625631 | 18 | 45517785 | C | A | -2.73 | 0.01 | 0.07 |  |
| rs6566073 | AX-13393772 | 18 | 75321417 | T | C | 2.31 | 0.01 | 0.08 |  |
| rs12983238 | AX-32728039 | 19 | 389487892 | A | G | 3.45 | 0.02 | 0.02 |  |
| rs79386654 | AX-32816651 | 19 | 7260800 | A | G | 6.37 | 0.01 | 0.07 |  |
| rs2059815 | AX-40460539 | 19 | 48028281 | C | G | 2.69 | 0.01 | 0.06 |  |
| rs1017165 | AX-13483178 | 20 | 15557647 | C | T | 3.50 | 0.01 | 0.09 |  |
| rs12627651 | AX-40719701 | 21 | 43340723 | G | A | 2.56 | 0.01 | 0.08 |  |
| rs17730978 | AX-33595265 | 22 | 30358377 | T | C | 9.18 | 0.05 | 0.00 |  |

Note: SNP: single nucleotide polymorphism; Prob_ID: Probeset ID (polymorphism identification in the microarray); Cro: Chromosome; Ref. Allele: Reference allele; Alt. Allele: Alternative allele; SBP: systolic blood pressure; DBP: diastolic blood pressure; ***: SNPs without rs identification.
